# Supplementary material for: To Apply or Not to Apply: A Survey Analysis of Grant Writing Costs and Benefits
Source: PLoS One. 2015 Mar 4;10(3):e0118494. doi: 10.1371/journal.pone.0118494 (PMC4349454; doi:10.1371/journal.pone.0118494)
Supplement: S1 Survey — (PDF) [file pone.0118494.s001.pdf]

Grants

Since January 2009, have you applied for a grant from NIH?

- Yes
- No

Since January 2009, have you applied for a grant from NSF?

- Yes
- No

Since January 2009, have you applied for a grant from NASA unrelated to new telescope (Hubble, Spitzer, Chandra, etc.) observations?

- Yes
- No

If you have submitted to any of the above agencies since January 2009, which agency did you most recently submit a grant to as a Principal/Primary Investigator?

- NIH
- NSF
- NASA
- I have not submitted a grant to any of these agencies as a PI

No grants 1

Since 2009, have you applied for a competitive externally funded grant?

- Yes
- No

No grants 2

Where did you submit your latest grant?

No grants 3

We are interested in why some people apply for grant funding as a Principal/Primary Investigator while others do

not. Are there any reasons why you have not applied for a grant?

Grants 1

For each of the questions below, please answer with regard to the last grant you applied for at ${q://QID4/ChoiceGroup/SelectedChoices}.

What year did you apply for this grant?

Please provide an estimate of how many total hours you spent preparing this proposal. This estimate should include background reading, data analyses, writing the proposal, preparing the budget, generating letters of support from department heads or other administrators, reading the funding agency documentation, etc. In short, this estimate should include any grant related activity.

Please enter your response in hours

Please estimate the number of hours all the other investigators combined spent working on this ${q://QID4/ChoiceGroup/SelectedChoices} submission.

Please enter your response in hours

How much money did you request in this grant application?

Please enter your response in dollars

Did this grant proposal receive funding from ${q://QID4/ChoiceGroup/SelectedChoices}?

- Yes
- No
- Not yet known
- The project was rated as fundable, pending budget approval

If the grant proposal was funded, how much money did you receive?

Please enter your response in dollars

Using the list below, please identify what discipline best describes the topic of this grant submission.

Below is a list of potential benefits that people may gain from applying for grants (whether or not the application is funded). Please indicate the degree to which you find each of these to be a benefit for you.

Writing a grant...

	Strongly disagree	Disagree	Somewhat disagree	Neither agree nor disagree	Somewhat agree	Agree	Strongly agree
Advances or fine-tunes my scientific thinking							
Enables me to consolidate or organize my research efforts/plans							
Helps me generate new ideas that I wouldn't have had otherwise							
Helps me plan the workflow for my research group							
Helps train/educate my graduate students and/or post-docs							
Helps me develop new collaborations							
Helps me focus on the big picture rather than just the details of my projects							
Results in text that I can then use for future papers and/or conference submissions							
For me, there are no benefits to grant writing except getting the grant!							

Grants 2

For the following questions we are interested in grant applications in which the outcome is already known.

In the years 2009, 2010 and 2011, how many grant applications did you submit to the NIH?

How many of your applications to the NIH were funded?

In the years 2009, 2010 and 2011, how many grant applications did you submit to the NSF?

How many of your applications to the NSF were funded?

In the years 2009, 2010 and 2011, how many grant applications did you submit to NASA unrelated to new telescope (Hubble, Spitzer, Chandra, etc.) observations?

How many of your applications to NASA were funded?

Demographics

What is your age?

Please enter your age in years

What is your gender?

- Male
- Female

Please indicate your current salary by converting it to a 12-month equivalent assuming you are fully funded.

- 50,000 or less
- 51,000 - 60,000
- 61,000 - 70,000
- 71,000 - 80,000
- 81,000 - 90,000
- 91,000 - 100,000
- 100,000 - 120,000
- 121,000 - 140,000
- 141,000 - 160,000
-

- 161,000 - 180,000
- 181,000 - 200,000
- Greater than 200,000

Which of the following best describes your current position?

- PhD Student
- Post-doc
- Assistant Professor
- Associate Professor
- Professor
- Emeritus Professor
- Research Assistant Professor or equivalent
- Research Associate Professor or equivalent
- Research Professor or equivalent
- Other (please specify)

Which of the following best describes the institution where you are working?

How long have you been employed at your current institution?

Please enter your response in years

In what year did you earn your PhD or equivalent?
